# Bioactive Compounds and Probiotics Mitigate Mastitis by Targeting NF-κB Signaling Pathway

**DOI:** 10.3390/biom14081011

**Published:** 2024-08-15

**Authors:** Muhammad Zahoor Khan, Liangliang Li, Tongtong Wang, Xiaotong Liu, Wenting Chen, Qingshan Ma, Muhammad Zahoor, Changfa Wang

**Affiliations:** 1 Liaocheng Research Institute of Donkey High-Efficiency Breeding and Ecological Feeding, Liaocheng University, Liaocheng 522000, China; 2 Department of Molecular Medicine, Institute of Basic Medical Sciences, University of Oslo, Sognsvannsveien, 90372 Oslo, Norway

**Keywords:** mastitis, mammary epithelial cells, TLRs, NF-κB signaling pathway, proinflammatory cytokines, probiotics, bioactive compounds

## Abstract

Mastitis is a significant inflammatory condition of the mammary gland in dairy cows. It is caused by bacterial infections and leads to substantial economic losses worldwide. The disease can be either clinical or sub-clinical and presents challenges such as reduced milk yield, increased treatment costs, and the need to cull affected cows. The pathogenic mechanisms of mastitis involve the activation of Toll-like receptors (TLRs), specifically TLR2 and TLR4. These receptors play crucial roles in recognizing pathogen-associated molecular patterns (PAMPs) and initiating immune responses through the NF-κB signaling pathway. Recent in vitro studies have emphasized the importance of the TLR2/TLR4/NF-κB signaling pathway in the development of mastitis, suggesting its potential as a therapeutic target. This review summarizes recent research on the role of the TLR2/TLR4/NF-κB signaling pathway in mastitis. It focuses on how the activation of TLRs leads to the production of proinflammatory cytokines, which, in turn, exacerbate the inflammatory response by activating the NF-κB signaling pathway in mammary gland tissues. Additionally, the review discusses various bioactive compounds and probiotics that have been identified as potential therapeutic agents for preventing and treating mastitis by targeting TLR2/TLR4/NF-κB signaling pathway. Overall, this review highlights the significance of targeting the TLR2/TLR4/NF-κB signaling pathway to develop effective therapeutic strategies against mastitis, which can enhance dairy cow health and reduce economic losses in the dairy industry.

## 1. Introduction

Dairy cow mastitis is a condition characterized by inflammation and a weakened immune response in the mammary gland tissue [1,2]. This occurs when harmful microorganisms invade the gland, causing damage and impairing milk production. In severe cases, affected cows may need to be culled, leading to substantial economic losses for the dairy industry. Multiple studies have shown that mastitis poses significant challenges, including decreased milk yield, the need for treatment, reproductive issues, and the necessity of culling [3,4,5,6,7]. Globally, mastitis results in an estimated annual economic cost of between US$19.7 billion and US$32 billion. In the United States, the annual economic loss due to mastitis is around US$2 billion, while in Canada, the dairy industry faces a Can$400 million loss (equivalent to US$318 million). China experiences annual fiscal losses ranging from 15 to 45 billion Chinese Yuan (CNY) due to mastitis [8,9,10].

The multifaceted nature of mastitis as a disease is widely recognized in the scientific community [11]. Mastitis is typically classified into clinical and sub-clinical forms. Clinical mastitis is characterized by pronounced pathological and physiological changes in mammary gland tissues, while sub-clinical mastitis, particularly when caused by *Staphylococcus aureus*, often presents more subtly, with no obvious symptoms except for elevated milk somatic cell counts and a decrease in milk yield [12,13,14,15,16,17]. The primary bacterial pathogens associated with mastitis include *Escherichia coli*, *Streptococcus uberis* (*S. uberis*), *S. dysgalactiae*, and *S. aureus* [18,19,20]. Toll-like receptors (TLRs) are considered the important pathogen sensors of Pattern recognition receptors (PRRs) [21,22]. Toll like Receptors are the major sensors stimulated during bacterial infection that begins the inflammatory process first by sensing pathogen-associated–molecular-patterns (PAMPs). The PAMPs then start to attract the immune cells towards the site of infection [23,24].

Among TLRs, TLR2 and TLR4 are particularly important in bacterial induced mastitis [25,26,27,28]. Notably, the TLR4 is more specifically utilized by LPS produced bacteria [29,30,31], while TLR2 are predominantly utilized by *S. aureus* and other gram-positive bacteria to induced inflammatory changes [32,33,34,35]. Activation of PRRs (i.e., TLR2 and TLR4) stimulates Nuclear Factor kappa-light-chain-enhancer of activated B cells (NF-κB) and Mitogen-activated protein kinase (MAPK) signaling pathways, thereby triggering the production of proinflammatory cytokines [36]. NF-κB is a family of key transcription factors that translocate to the nucleus in response to the activation of this signaling pathway. It is one of the major signaling pathways, transmitting pro-inflammatory response during infection and inflammation, as well as tumor growth and metastasis [37,38]. Under steady state, NF-κB is retained in the cytosol by a family of inhibitory proteins, such as IκB. However, upon stimulation, IKK is phosphorylated and degraded; therefore, the free NF-κB is translocated to the nucleus to induce the expression of its target genes. NF-κB is activated via two signaling pathways: the canonical and non-canonical (or alternative) signaling pathways. The canonical signaling pathway is activated by a broad range of stimuli, including TLRs, which induces phosphorylation of IκB and triggers its degradation by the proteasome [39]. On the other hand, the non-canonical signaling pathway responds to selective stimuli and does not depend on the degradation of IκB, but rather relies on the processing of the NF-κB precursor protein p100 [40]. During the processing of p100, its C-terminal IκB-like structure undergoes degradation, thereby triggering the nuclear translocation of mature p100 [41,42]. TLRs trigger the NF-κB pathways, further activating the proinflammatory cytokine cascade. The production of proinflammatory cytokines (TNF-α, IL-1β, and IL-6) is particularly increased in the infected mammary gland [43,44,45,46].

Bovine mastitis is commonly treated with antibiotics, but the excessive and indiscriminate use of antibiotics may contribute to antimicrobial resistance [47,48]. In order to combat this resistance, researchers are exploring alternative options to prevent bovine mastitis [48,49,50,51,52,53]. Recent studies have identified several bioactive compounds and probiotics that show promise in preventing and treating mastitis [35,41,54,55,56,57,58]. These compounds have been found to regulate the expression of key signaling molecules involved in the inflammatory response of mammary gland cells. More specifically, they appear to inhibit the activation of the NF-κB and TLRs signaling pathways [41,55,58], which play a crucial role in initiating and propagating inflammation in the mammary gland. These inflammatory processes are recognized as the main pathological mechanisms in mastitis development. Based on available data, this review aims to summarize the research progress on the critical role of TLR2/TLR4/NF-κB signaling pathway activation in the prevention and treatment of mastitis. Additionally, it delves into the details of how probiotics and bioactive compounds can mitigate mastitis by targeting the NF-κB signaling pathway.

## 2. Methodology

This review article was completed based on published findings related to activated TLR2/TLR4/NF-κB signaling pathway role in mastitis prevention research in last four years (April 2020–April 2024). The major searching esteemed academic databases data basis used for this review article were “Google Scholar, Web of Science, X-MOL, and PubMed”. The selection of literature was guided by a set of predetermined keywords: “Proinflammatory cytokines”, “Mammalian Mammary gland Cells”, “TLR2/TLR4/NF-κB Signaling pathways”, “Bioactive compounds”, ‘’Probiotics” and “Bacterial Induced mastitis”. To ensure the credibility and relevance of the sourced information, only articles published in English and indexed in Science Citation Index (SCI) Journals were considered for this review. In addition, book chapters and articles published in non-English languages were excluded from this review to maintain a focused and high-quality dataset for analysis.

## 3. Role of TLR2/TLR4/NF-κB Signaling Pathways in Mastitis Pathogenesis

### 3.1. TLRs Role in Mastitis Pathogenesis

TLRs, are essential sensors that detect pathogens through pattern recognition receptors (PRRs) [21,22]. Among these sensors, Toll-like receptors play a prominent role in initiating the inflammatory process during bacterial infections by recognizing PAMPs. This recognition leads to the mobilization of immune cells to the site of infection [23,24]. TLRs are composed of multiple leucine-rich repeat (LRR) sequences, which can be divided into three domains: the ectodomain, responsible for pathogen recognition; the transmembrane domain, facilitating localization; and the TIR domain, which recruits different downstream signaling adaptors. There are total of ten TLRs that have evolved and are characterized based on their ability to detect different pathogen loads in livestock. Based on their cellular localization, TLRs can be classified into two groups. The first group (TLRs 1, 2, 4, 5, and 6) is found on the cell membrane, while the second group (TLRs 3, 7, 8, and 9) is located in intracellular endosomes. Upon exposure to pathogens, all TLRs activate the myeloid differentiation primary response 88 (MyD88) signaling pathway, except for TLRs 3 and 4, which recruit the TRIF signaling pathway [33].

TLRs play a crucial role in immune system by recognizing pathogens and activating immune responses. NF-κB is a transcription factor that regulates the immune response to infection. Activation of NF-κB leads to the transcription of pro-inflammatory genes, including cytokines, chemokines, and adhesion molecules. Recently a study reported the polymorphisms in TLR4 gene on promoter and 5′ untranslated region (5′UTR) were significantly associated with mastitis in cattle mammary gland tissue [30]. Consistently another study documented that SNPs at 5′UTR (c.1-g27) and coding region (c.87 C>A, c.575 A>G, and c.576 T>G were responsible for mastitis susceptibility in Egyptian buffalo breed [29].

### 3.2. NF-κB Signaling Pathways Role in Mastitis Pathogenesis

Upon recognition of specific stimulant, TLR2 and TLR4 undergo conformational changes and initiate downstream signaling cascades. Both TLR2 and TLR4 activate a common adaptor protein called MyD88, which recruits and activates IRAKs (interleu-kin-1 receptor-associated kinases), leading to the activation of TNF receptor-associated factor 6(TRAF6). This results in the activation of the IKK complex (IκB kinase), which phosphorylates IκB, the inhibitor of NF-κB. The phosphorylation of IκB by the IKK complex, target it for proteasomal degradation and thereby allowing NF-κB translocation to the nucleus. TLR4 also stimulate the non-canonical NF-κB signaling pathway in latent stage during persistent infection and inflammation [40]. In the nucleus, NF-κB induces the expression of pro-inflammatory genes, leading to the production of cytokines, chemokines, and other inflammatory mediators. The activation of TLR2 and TLR4 can have synergistic effects, amplifying the inflammatory response and ensuring a robust and rapid response to a wide range of pathogens. However, excessive activation and crosstalk can lead to an overproduction of inflammatory mediators, contributing to the pathology of mastitis.

**Figure 1:**
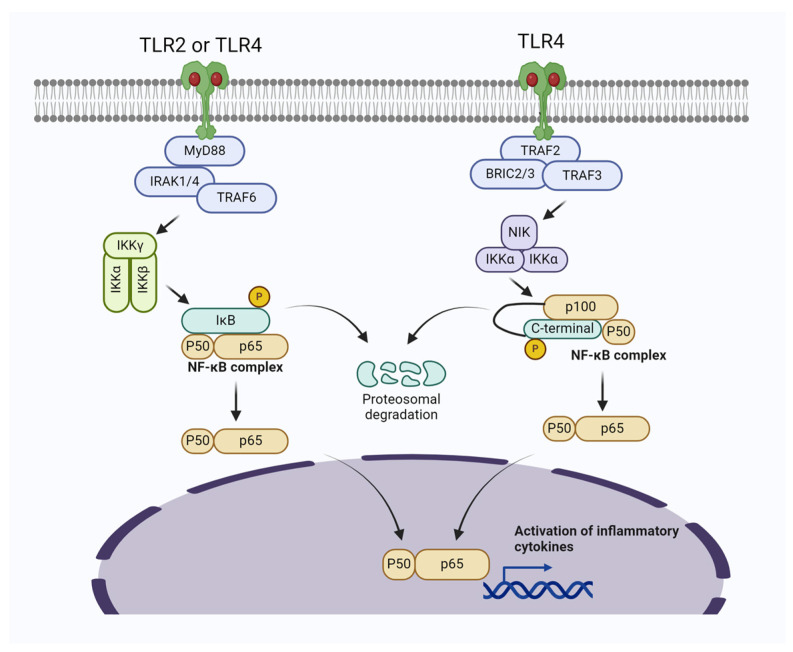
Bacterial induced mastitis via activating TLR2/TLR4/NF-κB signaling pathways in mammary gland cells. The activation of Myeloid differentiation primary response 88 (MyD88)-dependent signal transduction initiates the recruitment of IRAK1/4 and TRAF6, subsequently leading to the activation of the TAK1-IKK complex. This complex then triggers the activation of NF-κB. In the noncanonical NF-κB pathway, specific subsets of the TNFR superfamily interact with their ligands, resulting in the recruitment of TRAF2/3. Following the ubiquitination and degradation of TRAF3, NF-κB-inducing kinase (NIK) accumulates in the cytoplasm and associates with IKKα, promoting the phosphorylation of p100.

Negative feedback mechanisms exist to prevent excessive inflammation. For instance, certain cytokines produced in response to NF-κB activation can inhibit further TLR signaling. Crosstalk also involves interactions with other signaling pathways, such as those mediated by cytokines like IL-6 and TNF-α, which can further modulate the immune response. Upon infection, TLR2 and TLR4 are activated, leading to NF-κB activation and a strong inflammatory response (Figure 1). This results in the production of cytokines, chemokines, and other inflammatory mediators that help fight off the infection but also cause tissue damage and symptoms of mastitis. If the infection is not resolved, persistent activation of these pathways can lead to chronic inflammation, tissue remodeling, and fibrosis, contributing to long-term damage to the mammary gland.

Molecular mechanisms have revealed that the NF-κB signaling pathway plays a key role in the pathogenesis of mastitis in mammary gland cells [2,57]. In line with this, a study reported elevated phosphorylation of MAPKs and AKT/NF-κB P65 signaling pathways, resulting in upregulation of IL-6 and IL-1β and subsequent inflammatory changes in mouse mammary gland cells [41]. Similarly, Akhter et al. [58] found that *S. aureus* upregulates the expressions of TLR2, TLR4, p-65 IκBα, TNF-α, IL-1β, and IL-6, leading to the activation of NF-κB/MAPKs pathways in bovine mammary epithelial cells (BMECs), which ultimately results in mastitis. Other studies have also shown that pathogenic bacteria induce inflammatory changes and mastitis through the activation of NF-κB in the mammary gland [59,60]. It is worth noting that during pathogen-induced mastitis, there is an increase in reactive oxygen species (ROS) levels, oxidative stress, apoptosis of cells, and significant inflammatory changes. Furthermore, these changes are associated with the upregulation of the NF-κB signaling pathway and the prevention of the activation of the Nrf2/HO-1 pathway [59].

By using transcriptomic analysis, Liu J et al. reported several key signalings including NF-κB signaling pathway in LPS infected BMECs [2]. Similarly, a methylomic screening of milk somatic cell counts from infected cattle’s udder revealed extensive methylation of genes involved in the activation of the NF-κB signaling pathway [61]. Consistently other studies reported that exosomal miR-155 and miR-223 inhibitors downregulate the expression of TLR2, TLR4, TNF-α, and IL-1β, along with the suppression of phosphorylation NF-κB signaling pathway [26,62,63]. The summary of study highlighting the role of TLR2/TLR4/NF-κB signaling pathways in mastitis pathogenesis has been provided in Table 1.

## 4. Role of Bioactive Compounds and Probiotics Targeting TLR2/TLR4/NF-κB Signaling Pathways in Mastitis Mitigation

Despite efforts to prevent and treat mastitis, it remains the most common health problem in dairy herds. Therefore, there has been an increasing interest in exploring alternative therapeutic procedures as a substitute for traditional treatment [69,70]. In recent years, there has been a significant rise in research dedicated to finding non-antibiotic alternatives for managing bovine mastitis [47]. The excessive and incorrect use of antibiotics in treating mastitis has caused bacteria strains to become resistant to antibiotics. This resistance can make antibiotics less effective or completely ineffective, which is a major challenge in treating mastitis. Moreover, using antibiotics to treat mastitis in dairy cows can result in antibiotic residues in milk, which raises concerns about food safety. These residues not only pose health risks to consumers but also lead to milk disposal, causing economic losses. Therefore, it is important to explore alternative treatments. Probiotics and bioactive compounds offer promising alternatives that do not contribute to antibiotic resistance. In addition, these beneficial substances do not leave harmful residues, resulting in the production of safer milk and reducing the economic impact. It has been demonstrated that probiotics and bioactive compounds have the ability to modulate the TLR2/TLR4/NF-κB pathway, potentially reducing the inflammatory response and aiding in the prevention of mastitis.

### 4.1. Bioactive Compounds Targeting TLR2/TLR4/NF-κB Signaling Pathways in Mastitis Mitigation

**Figure 2:**
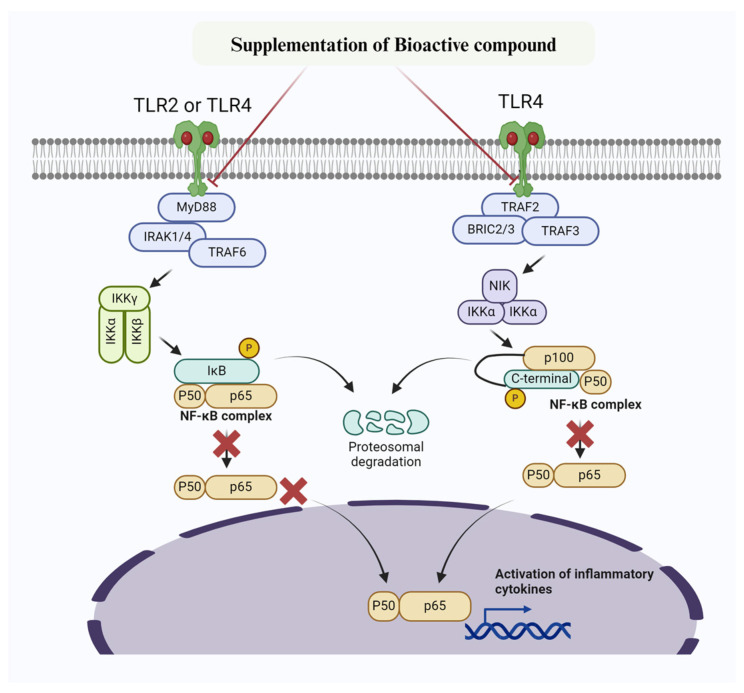
Mechanism of bioactive compounds targeting NF-κB signaling pathway to prevent inflammatory changes and mastitis. Bioactive compounds inhibit IKK, thereby preventing phosphorylation of IκBα. Furthermore, IκBα super-repressor specifically prevent NF-κB subunits p65 and p50 and other members from entering the nucleus.

Extensive research has been conducted on the potential of bioactive compounds to mitigate mastitis by reducing inflammation and fighting against microbes (Table 2, Figure 2) [71,72]. Consistently, Sodium butyrate has been shown to have positive effects in reducing oxidative stress induced by LPS. This includes decreasing ROS and MDA levels, increasing the activity of SOD, GSH-Px, and CAT, as well as reducing inflammation by lowering IL-6, IL-1β, and TNF-α levels. Additionally, Sodium butyrate has demonstrated its anti-apoptotic properties by reducing caspase and Bax levels, while increasing Bcl-2 levels [73]. Sodium butyrate also inhibits NF-κB and caspase/Bax signaling pathways and increases the expression of Nrf2, Keap1, NQO-1, and HO-1 in bovine mammary epithelial cells [73]. Lipoteichoic acid (LTA), a bacterial endotoxin found in the cytoderm of *S. aureus*, contributes to inflammatory changes associated with to mastitis. Arab et al. [74] have demonstrated that metformin significantly suppresses the expression of NF-κBp65, cyclooxygenase 2 (COX2), IL-1β, and IL-6 in BMECs when exposed to LTA. Furthermore, they found that metformin enhanced the levels of AMPK, Nrf2, HO-1, and Gpx1, thereby promoting the antioxidant response in BMECs [74]. Overall, their study concludes that metformin prevents inflammatory changes and mastitis by activating the AMPK/Nrf2/NF-κB signaling pathway in BMECs [74]. Similarly, another study reported that Brazilin (isolated from the traditional herbal medicine *Caesalpinia sappan* L.) suppressed TLR2, TNF-α, IL-1β, and IL-6 levels by inhibiting the TLR2/NF-κB/MAPK signaling pathways in mammary gland tissues, thus preventing *S. aureus*-induced mastitis [75]. A recent study found that daidzein significantly alleviated LPS-induced mastitis in mouse mammary gland epithelial cells by inhibiting IL-6 and IL-1β levels and suppressing the activation of MAPK/NF-κB signaling pathways [76]. Furthermore, it has been documented that diosmetin relieved *S. aureus*-induced mastitis in mice by inhibiting the expression of MPO, TNF-α, IL-1β, and NF-κB [76]. In addition, diosmetin enhanced the antioxidant response by upregulating the levels of glutathione (GSH), glutathione peroxidase 4 (GPX4), sirtuin 1 (SIRT1), nuclear factor erythroid2-related factor 2 (Nrf2), and heme oxygenase 1 (HO-1), which were decreased by *S. aureus* in in mouse mammary epithelial cells (MMECs) [76]. It has also been demonstrated that *Abrus cantoniensis* total flavonoids (ATFs) can prevent lipopolysaccharide-induced mastitis in mouse mammary gland cells. This is achieved by enhancing gut microbiota and inhibiting the expression of the CD14/TLR4/NF-κB/MAPK pathway [77]. In addition, they noticed significant suppression of TNF-α, IL-1β, and IL-6 in response to ATFs [77]. Similarly, another study found that pedunculoside, a bioactive component of Aquifoliaceae, prevents LPS-induced mastitis and maintains the integrity of the blood-milk barrier. This is achieved through downregulation of the AKT/NF-κB/MAPK signaling pathways [78]. Furthermore, the study explored the reversal of inflammatory changes in MMECs through suppression of INF-α, IL-6, IL-1β, MPO, and iNOS levels [78]. Li et al. [79] revealed that jingfang (JF) granules also prevent LPS-induced mastitis. This is accomplished by reducing MPO activity and inhibiting the expression of IL-1β, IL-6, and TNF-α. Additionally, JF treatment downregulates TLR4, P-NF-κB, NLRP3, ASC, Caspase-1, IL-1β, and MAPK signaling pathways. To maintain the integrity of the blood-milk barrier, JF treatment also promotes the expression of ZO-1, claudin-3, and occludin [79]. Evodiamine [80] and oxymatrine [81], among other bioactive compounds, have also been reported to prevent mastitis in MMECs induced by LPS. They achieve this through the suppression of AKT/NF-κB p65/MAPK signaling pathways and pro-inflammatory cytokines (TNF-α, IL-1β, and IL-6). Similarly, rosmarinic acid and nuciferine alleviate LPS-induced mastitis by inhibiting the TLR4/MyD88/NF-κB signaling pathway and production of TNF-α, IL-1β, and IL-6 in MMECs [82,83]. Studies have reported that leonurine [84] and magnolol [85] suppress pro-inflammatory cytokines (TNF-α, IL-1β, and IL-6), iNOS, COX-2, and TLR4, while enhancing IL-10 levels. This is followed by the downregulation of NF-κB-p38 and TLR4/NF-κB/MAPK signaling to mitigate mastitis in MMECs. Gambogic acid (GA) was also reported to alleviate LPS-induced mastitis by reducing IL-6, TNF-α, and IL-1β levels. This is achieved through inhibiting the phosphorylation of the NF-κB/MAPK pathway in MMECs [86]. Furthermore, other bioactive compounds (sodium propionate, curcumin, hederacoside-C, palmatine, sodium butyrate, allicin, oligomeric proanthocyanidins, and dehydroandrographolide) have been reported to inhibit the NF-κB/MAPK signaling pathways and pro-inflammatory cytokines to prevent mastitis [56,73,87,88,89,90,91,92,93]. Based on the existing findings, it has been concluded that bioactive compounds targeting the TLR2/TLR4/NF-κB signaling pathways show promise in mitigating mastitis. This is achieved by reducing inflammation and oxidative stress, as well as combating microbes. 

### 4.2. Role of Probiotics Supplementation in Targeting TLR2/TLR4/NF-κB Signaling to Mitigate Mastitis

Probiotics are live microorganisms that, when consumed in sufficient amounts, provide health benefits to the host [108]. They exert their positive effects through various mechanisms, such as modifying the composition of the local microbiota or directly acting on pathogenic microorganisms. Probiotics can adhere to and interact with epithelial cells, improving their barrier function and promoting cell renewal. Additionally, they can enhance the host’s immune system response [109,110]. Touza-Otero and colleagues have previously published a comprehensive review on the role of probiotics in relieving mastitis [47]. However, this study specifically examines the molecular mechanism of probiotics in preventing mastitis, with a particular emphasis on targeting the NF-κB signaling pathway.

**Figure 3:**
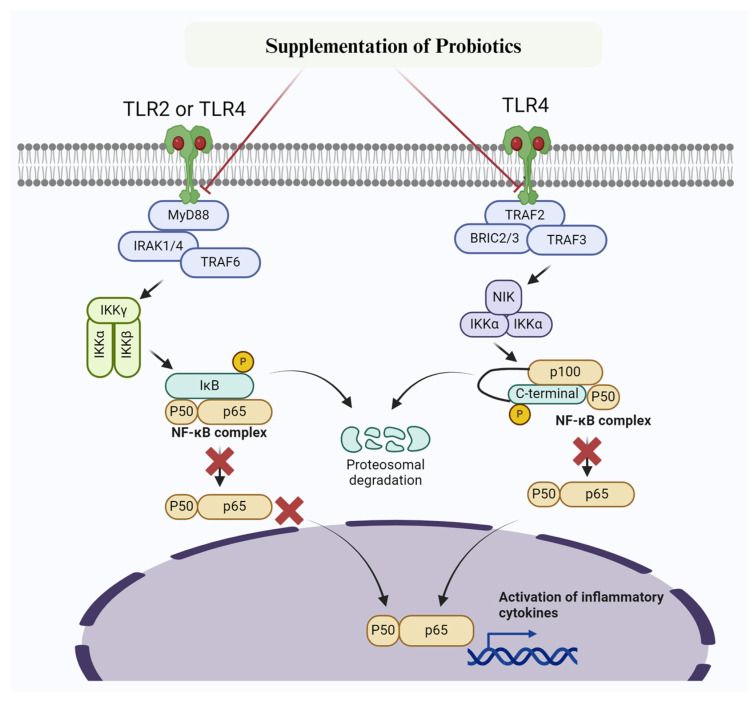
Mechanism of probiotic targetingTLR2/TLR4 /NF-κB signaling pathway to prevent inflammatory changes and mastitis. Probiotics inhibit IKK, thereby preventing phosphorylation of IκBα. Furthermore, IκBα super-repressor specifically prevent NF-κB subunits p65 and p50 and other members from entering the nucleus.

The anti-inflammatory effect of probiotics in several diseases, including mastitis, has been well-documented [111,112,113,114,115]. Consequently, the association of gut microbiota with mastitis has been well studied [116,117]. Consistently, several probiotics, such as *Lactobacillus plantarum* (*L. plantarum 17-5*) [118,119], *Lactobacillus casei* [120], *Lactobacillus rhamnosus GR-1* [114,121], and *Bacillus subtilis* [114,122], have been found to prevent inflammatory changes in MMECs and subsequently prevent mastitis caused by *E. coli.* Furthermore, *Bacillus subtilis* was found to prevent inflammatory changes in MMECs by inhibiting TNF-α, IL-1β, IL-6, and TLR4 and blocking the TLR4/NF-κB/MAPK signaling pathway [122]. Consequently, Li K et al. [118,119] reported that *Lactobacillus plantarum 17-5* and *Lactobacillus casei* attenuate *E. coli*-induced inflammatory changes by suppressing the NF-κB/MAPK signaling pathway, leading to decreased production of IL-1β, IL-6, and TNF-α in MMECs [119,121]. Furthermore, in their studies on *Lactobacillus plantarum KLDS 1.0344A*, Chen et al. [123] experimentally demonstrated its effectiveness in alleviating LPS-induced mastitis through in vitro (BMECs) and in vivo (mastitis mouse model) experiments. They discovered that pre-treatment with *Lactobacillus plantarum* KLDS 1.0344A significantly reduced the phosphorylated expression levels of p65 and IκBα in the NF-κB/MAPK signaling pathway, thereby controlling the expression of downstream inflammatory factors. Additionally, *Lactobacillus plantarum* KLDS 1.0344A specifically suppressed the expression of pro-inflammatory cytokines such as IL-6, IL-1β, and TNF-α [123]. Most mastitis-causing pathogens are known to inhibit lactic acid bacteria. However, it has been reported that *Lactobacillus plantarum* KLDS 1.0344 enhances lactic acid bacteria and reduces MPO activity, thereby mitigating mastitis [123]. Another study by Qiu et al. [124] found that the probiotic *Enterococcus mundtii H81* can prevent *S. aureus*-induced mastitis. It achieves this by inhibiting the activation of the NF-κB signaling pathway and improving the integrity of the blood-milk barrier. Furthermore, they discovered that *E. mundtii H81* suppressed the levels of TNF-α and IL-1β, MPO activity, and downregulated the phosphorylation of p65 NF-κB and IκB. It also elevated the expression of tight junction proteins claudin 3 and ZO-1, thus protecting the mammary gland from *S. aureus* infection [124]. The role of probiotics in mitigating mastitis by targeting NF-κB signaling pathway has been summarized in Figure 3.

## 5. Summary and Future Perspective

Currently, the primary approach to treating mastitis is through the use of antibiotics. Although antibiotics are effective in fighting bacterial pathogens, however, their extensive use has raised concerns regarding antibiotic resistance. The development of antibiotic resistance not only weakens the effectiveness of antibiotics over time but also poses a major risk to public health. Additionally, the presence of antibiotic residues in milk raises concerns about its safety, potentially posing health risks to consumers and creating regulatory complications for dairy producers.

Given these challenges, there is growing interest in alternative therapies, particularly bioactive compounds and probiotics. Bioactive compounds include various plant derivatives, peptides, and other natural molecules, which have shown potential in reducing inflammation and oxidative stress. These compounds can modulate immune responses, thereby helping manage the symptoms of mastitis without inducing antibiotic resistance. For instance, certain flavonoids and essential oils have demonstrated anti-inflammatory and antioxidant properties, capable of alleviating the symptoms of mastitis. Similarly, probiotics, as beneficial microorganisms, offer another promising alternative. These microorganisms can inhibit key inflammatory pathways, enhance the host’s immune response, and compete with pathogenic bacteria in the mammary gland. By maintaining a healthy microbial balance, probiotics can help prevent infections or reduce their severity. For example, certain strains of lactic acid bacteria and bifidobacteria have been shown to enhance mammary gland health and reduce the incidence of mastitis in dairy cows.

Although these alternative methods are promising, further research is needed to fully understand the exact mechanisms by which bioactive compounds and probiotics exert their beneficial effects. Detailed studies are necessary to determine the optimal doses, delivery methods, and combinations of these agents to maximize their efficacy. Additionally, large-scale clinical trials and field studies are required to confirm their safety and effectiveness in different dairy production systems. Future research should focus on elucidating the molecular and cellular pathways affected by these alternative treatments.

Bioactive compounds and probiotics may exert their effects by modulating the TLR2/TLR4/NF-κB signaling pathways. They may influence TLR2/TLR4/NF-κB signaling pathway by interacting with mammary immune cells, potentially enhancing anti-inflammatory responses and promoting tissue repair. By optimizing the use of bioactive compounds and probiotics, we can develop effective strategies for the prevention and treatment of mastitis, thereby reducing dependence on antibiotics. This shift not only addresses the issue of antibiotic resistance but also ensures the safety and quality of milk, benefiting both producers and consumers. Understanding and targeting the TLR2/TLR4/NF-κB signaling pathways will be a key part of this research, providing new insights into the mechanisms of mastitis and how alternative therapies can combat this common dairy disease. Additionally, studying other cellular signaling pathways involved in mastitis and their activation mechanisms in response to probiotics and bioactive compounds will contribute to a more comprehensive understanding of their roles and potential therapeutic targets.

## 6. Conclusions

The in vitro investigations discussed in this review strongly support the idea that the TLR2/TLR4/NF-κB signaling pathway plays a crucial role in the development of mastitis in dairy cows. When this pathway is activated, it triggers the production of pro-inflammatory cytokines, which worsen the inflammatory response in the mammary gland tissues. By targeting the TLR2/TLR4/NF-κB signaling pathway, we can potentially find a promising therapeutic approach to reduce mastitis. The review also highlights the potential of various bioactive compounds and probiotics to prevent and treat mastitis by influencing this signaling pathway. Utilizing probiotics and bioactive compounds to mitigate mastitis could be the optimal alternative to decrease antibiotic usage and combat the growing problem of antibiotic resistance, which poses a serious threat to public health. These findings emphasize the need for further research on the TLR2/TLR4/NF-κB signaling pathway to improve dairy cow mammary gland health and minimize economic losses in the dairy industry. In addition, future studies must consider the specific sites of action of bioactive compounds in regulating the TLR2/TLR4/NF-κB signaling pathway. Understanding these mechanisms is crucial for the further development of potential therapeutic molecules.

## Figures and Tables

**Table 1:** Bacterial activated TLRs/NF-κB signaling pathways role in mastitis pathogenesis.

Causative Agent	Targeted Signaling Pathway	Biological Functions	Types of Cells	References
*Mycoplasma bovis*-induced mastitis	TLR2/TLR4/NF-κB signaling pathway	⟡ Elevated expression of interleukin (IL)-1β, IL-6, IL-8 and tumor necrosis factor (TNF)-α followed by inflammatory changes in mammary epithelial cells	BMECs	[64,65]
*Lactococcus garvieae* induced mastitis	TLR2/NLRP3/NF-κB pathway	⟡ Promoted the expression of TLR2/NLRP3 followed by regulation of NF-κB in mice mammary gland cells. ⟡ Enhanced inflammatory changes mammary epithelial cells.	Mouse Mammary epithelial cells (MMECs)	[57]
*S. uberis*-induced mastitis	TLR2	⟡ Upregulated the levels of TLR2 and TNF-α followed inflammatory changes in milk-isolated white blood cells (mWBCs)	Milk-isolated white blood cells (mWBCs)	[66]
*S. aureus*-induced mastitis	TLR2, TLR4, and NLRP3	⟡ Elevated expressions of TLR2, TLR4, IL-1β, IL-6, and NLRP3 ⟡ Increased level of prostaglandin E2 (PGE2) ⟡ Inflammatory changes in BMECs	BMECs	[67]
Lipoteichoic acid (LTA)-induced mastitis	TLR2/NF-κB signaling pathways	⟡ Upregulated the expression of TLR2 followed by increased inflammatory changes in BMECs via elevated levels of proinflammatory cytokines	BMECs	[68]
*S. aureus*-induced mastitis	NF-κB signaling pathways	⟡ LPS induced mastitis via upregulation of the expression exosomal miR-193b-5p followed by activation of NF-κB signaling pathway in dairy cow mammary alveolar (Mac-T) cells	Mac-T cells	[63]

**Table 2:** Summary of bioactive compounds targeting TLR2/TLR4/NF-κB signaling pathways in mastitis mitigation.

Bioactive Compounds	Targeted Signaling Pathway	Biological Functions	Types of Cells	References
Caffeic acid	NF-κB/Nrf2 signaling pathway	⟡ Significantly suppressed the LPS-induced ROS production ⟡ Increased the expression of Nrf2 and reduced the NF-κB level to enhance antioxidant response and suppressed inflammation ⟡ Relieved mammary tissue damage and inhibited the oxidative burst and neutrophil chemotaxis	MMECs	[94]
Jiawei Yanghe decoction (JWYHD)	TLR4/Myd88/NF-κB signaling pathway	⟡ Decreased the MYD88, TLR4, IL-1β, IL-6, IL-8, TNF-α, IκB, and the p65 expressions followed by inhibition of inflammatory changes in mice mammary epithelial cells	BMECs	[95]
Chlorogenic acid	NF-κB/Nrf2/HO-1 signaling pathway	⟡ The inflammatory changes were suppressed by reducing the expressions of NF-κB, IL-6, IL-8, TNF-α, IL-1β, and iNOS	BMECs	[38]
Resveratrol	NF-κB signaling pathway	⟡ Decreased the expression of NF-κB, P65 and P65 and Nrf2 Expression ⟡ Relieved inflammatory changes and oxidative stress in mammary gland cells	MMECs	[96]
Maslinic acid (Extracted from Olives)	AKT/NF-κB/MAPK signaling pathway	⟡ Suppressed expression of NLRP3, IL-6, IL-1β, and TNF-α in LPS-induced mastatic MMECs ⟡ Protects the blood-milk barrier by maintaining tight junction proteins expression ⟡ Prevents udder infection	MMECs	[97]
Quyu Xiaozhong recipe	TLR4/NF-κB signaling pathway	⟡ Inhibited the expressions of TLR4, NF-κB-p65, and IκB-α ⟡ Prevented inflammatory changes induced by *S. aureus* in rat’s mammary gland cells	Rat’s MECs	[98]
Wogonin	NF-κB /Nrf2/HO-1 signaling pathway	⟡ Inhibited inflammation (NF-κB, TNF-α and IL-1β) and enhanced the antioxidant response (increased the Nrf2, HO-1, GSH, SOD and decreased MDA levels) in LPS treated MMECs	MMECs	[55]
Hexadecanamide	NF-κB pathway	⟡ Relieved LPS-induced inflammatory changes and maintain the blood barrier integrity of MMECs ⟡ Restrict the pathogenesis of mastitis	MMECs	[99]
Retinoic acid	NF-κB/NLRP3 signaling pathway	⟡ Inhibit low-grade endotoxemia-induced mammary injury and proinflammatory cytokines production following mitigation of mastitis by downregulating NF-κB/NLRP3 signaling pathway in MMECs ⟡ Maintain blood-milk barrier integrity via upregulation of ⟡ tight junction proteins, including ZO-1, occludin and claudin-3	MMECs	[100]
Guaiacol (2-Hydroxyanisole phenol, 2-methoxy), and ethyl ferulate/ethyl 4′-hydroxy-3′methoxycinnamate extraction of *Curcuma longa*	TLR4/NF-κB signaling pathway	⟡ Suppressed the expression of TNFα, TLR4, and IL-6 in LPS-induced mastitic buffalo mammary epithelial cells ⟡ Enhance the anti-inflammatory and antioxidative responses in buffalo mammary gland cells	Buffalo MECs	[101]
Jingfang Granules	NF-κB/NLRP3/MAPK signaling pathways	⟡ JF prevent LPS-induced mastitis by reducing ⟡ the MPO activity, inhibition of the expression ⟡ of pro-inflammatory factors (IL-1β, IL-6 and ⟡ TNF-α), NLRP3, MAPK, TLR4, and phosphorylation levels of NF-κB and p38 ⟡ In addition, the JF improved the integrity of ⟡ the blood-milk barrier by increasing the expression of ZO-1, Claudin-3 and Occludin.	MMECs	[79]
Forsythiaside A	NF-κB/MAPK signaling pathway	⟡ Reduced the IL-1β, IL-6, TNF-α, p38 MAPK, IκBα, and NF-κB p65 levels in LPS treated mice mammary epithelial cells	MMECs	[102]
*Taraxacum mongolicum*	TLR2/NF-κB/MAPKs pathways	⟡ Prevents S. aureus induced mastitis by suppressing TNF-α, IL-6 and IL-1β levels and inhibited the MPO activity ⟡ Decreased expression of TLR2, and the phosphorylations of inhibitor κBα (IκBα), p65, p38	MMECs	[35]
Metformin	AMPK/NF-κB/NLRP3 signaling pathway	⟡ Suppressed the LPS-induced levels of IL-1β, TNF-α, IL6, CXCL8, MYD88, NLRP3, Caspase1, ASC and TLR4 ⟡ Decreased the phosphorylation of NF-κB p65 and IκBα ⟡ Enhanced the expression of AMPK ⟡ Prevented LPS induced inflammatory changes and apoptosis in BMECs	BMECs	[103]
Evodiamine	AKT/NF-κB p65/MAPK Signaling Pathways	⟡ Suppressed the pro-inflammatory cytokines production ⟡ Inhibit the activation of phosphorylation of AKT, NF-κBp65, and MAPK in MMECs to prevent mastitis	MMECs	[80]
Curcumin	TLR2/NF-κB Signaling Pathways	⟡ Reduced LPS-induced S. aureus induced inflammatory changes via suppression of IL-1β, IL-6, and TNF-α, and inhibition of TLR2/NF-κB signaling Pathways MMECs	MMECs	[34]
Morin	AKT/NF-κB p65/MAPK/NLRP3 Signaling Pathways	⟡ Inhibited the activation of AKT/NF-κB p65/MAPK/NLRP3 Signaling Pathways induced by LPS in MMECs ⟡ Suppressed the expressions of MPO, IL-1β, IL-6, and TNF-α ⟡ Protected the integrity of blood-milk barrier and prevented mastitis	MMECs	[104,105]
Vanillin	NF-κB/MAPK signaling pathways	⟡ Decreased the expression of IL-1β, IL-6, iNOS, and MPO activity in LPS-induced mastitis in MMECs in response to vanillin ⟡ The vanillin maintains the blood-milk barrier via upregulation of zona occludens (Zo-1), claudin-3, and occluding ⟡ Suppressed the activation of NF-κB/MAPK signaling pathways	MMECs	[36]
Shikonin (Bioactive naphthoquinone constituent extracted from Chinese herb *Lithospermum Erythrorhizon*)	NF-κB signaling pathway	⟡ Decreased level of proinflammatory cytokines (TNF-α, IL-1β, and IL-6) ⟡ Suppressed the phosphorylation levels of IκBα and p65 ⟡ Prevent LPS induced mastitis in MMECs	MMECs	[106]
Cynatratoside-C from Cynanchum atratum	TLR4/NF-κB/MAPK signaling pathways	⟡ Cynatratoside-C blocked the expression of TLR4 followed by inhibiting the activation of NF-Κb and MAPK ⟡ The proinflammatory cytokines (TNF-α, IL-1β, and IL-6) ⟡ Expressions were downregulated and enhanced antioxidant response by upregulating the levels of SOD and GPx and reduced MDA content ⟡ Protected MMECs from mastitis	MMECs	[107]
Nuciferine	TLR4/NF-κB signaling pathway	⟡ Inhibited the levels of MPO, TNF-α and IL-1β following suppression of TLR4/NF-κB signaling pathway to prevent inflammatory changes caused by LPS. ⟡ Protected MMECs from LPS-induced mastitis	MMECs	[83]

## Data Availability

All the data are included in the manuscript.
